# Improving Care for Patients with Chronic Hepatitis B via Establishment of a Disease Registry

**DOI:** 10.4269/ajtmh.21-1013

**Published:** 2022-06-13

**Authors:** Malini B. DeSilva, Ann Settgast, Ella Chrenka, Amy J. Kodet, Patricia F. Walker

**Affiliations:** ^1^ HealthPartners Institute, Bloomington, Minnesota;; ^2^ Department of Medicine, University of Minnesota, Minneapolis, Minnesota;; ^3^ HealthPartners Center for International Health, St. Paul, Minnesota;; ^4^ HealthPartners Travel and Tropical Medicine Department, St. Paul, Minnesota

## Abstract

In the United States, there is poor clinician adherence to the American Association for the Study of Liver Disease and other guidelines for chronic hepatitis B virus (CHB) management. This prospective cohort study evaluated whether a CHB registry improves CHB management. We included patients with CHB aged ≥ 18 years and who had a clinical encounter during September 1, 2016–August 31, 2019. We divided patients into three groups based on care received before September 1, 2019: 1) CIH: primary care clinician at HealthPartners Center for International Health, 2) GI: not CIH and seen by gastroenterology within previous 18 months, and 3) primary care (PC): not CIH and not seen by gastroenterology within previous 18 months. We created and implemented a CHB registry at CIH that allowed staff to identify and perform outreach to patients overdue for CHB management. Patients with laboratory testing (i.e., alanine transaminase and hepatitis B virus DNA) and hepatocellular carcinoma screening in the previous 12 months were considered up to date (UTD). We compared UTD rates between groups at baseline (September 1, 2019) and pilot CHB registry end (February 28, 2020). We evaluated 4,872 patients, 52% of whom were female: 213 CIH, 656 GI, and 4,003 PC. At baseline, GI patients were most UTD (69%) followed by CIH (51%) and PC (11%). At pilot end the percent of UTD patients at CIH increased by 11%, GI decreased by 10%, and PC was unchanged. CHB registry use standardized care and increased the percent of CHB patients with recent laboratory testing and HCC screening.

## INTRODUCTION

Hepatitis B, a liver infection caused by hepatitis B virus (HBV), remains a major global health problem despite safe, effective immunizations and antiviral treatments. The WHO’s goal to eliminate viral hepatitis by 2030 depends on increased vaccination, diagnosis, and linkage to care for individuals infected with HBV.^1^ Chronic hepatitis B (CHB) can cause cirrhosis, hepatocellular carcinoma (HCC), and death.^2^^–^^5^ Ongoing management including laboratory testing, antiviral therapy (if indicated), and liver imaging are necessary to decrease risk of CHB sequelae.^6^ There is poor clinician adherence to the American Association for the Study of Liver Disease (AASLD) and other guidelines for CHB management related to laboratory monitoring and HCC screening.^7^^–^^9^ There are also multiple barriers at the patient, clinician, and system levels that contribute to low uptake of evidence-based practices for HBV care,^10^^,^^11^ including low patient awareness of HBV infection.^12^ Improving adherence to guidelines is important to evaluate disease progression and treatment eligibility, which in turn may hasten treatment initiation resulting in fewer HBV infection complications.^7^

In the United States, the rate of new HBV infections has remained low since 1991 when HBV vaccine was added to the routine childhood immunization schedule.^13^ Prevalence of CHB among U.S.-born persons is extremely low, estimated at 0.1% to 0.2%.^14^ During 2018, 14,207 cases of chronic HBV infection were reported in the United States.^13^ The most common risk factor for CHB is birth in a country with ≥ 2% HBV infection prevalence.^15^ When the contribution of foreign-born persons is included, the estimated number of people with CHB in the United States may be as high as 2.2 million.^14^ Given the disproportionate burden of CHB among foreign-born populations in the United States, coupled with their unique language, cultural and structural barriers, improvement in CHB management represents an opportunity to decrease healthcare disparities.

Chronic disease registries, lists of patients with, or at-risk of developing, particular chronic illnesses, embedded within an EHR may facilitate identification of patients with certain conditions, track clinical quality metrics and outcomes, and improve coordination of care.^16^^–^^19^ We are unaware of any health systems with an EHR-integrated chronic disease registry to improve long-term management of CHB infected patients. We created a pilot hepatitis B registry with the goals of standardizing care and improving long-term follow-up for patients with CHB. We evaluated CHB management for patients included in the pilot registry compared with those in usual care in two key areas: laboratory monitoring and HCC screening.

## MATERIALS AND METHODS

### Setting/data source.

We conducted a prospective cohort quality improvement project including patients with CHB seen at HealthPartners (HP). The HP Institutional Review Board determined this project did not meet the definition of Human Subjects Research because it was a quality improvement project. HP is the largest consumer-governed nonprofit healthcare organization in the country, providing care to more than 1.2 million patients annually. HP Center for International Health is a primary care clinic that predominantly serves foreign-born patients with limited English proficiency. We implemented the pilot hepatitis B registry at HP Center for International Health during September 2019–February 2020. The hepatitis B registry was based on other chronic disease registries currently in use at HP. We used the EHR to identify patients who met registry inclusion criteria. Research staff collaborated with primary care teams to develop detailed registry workflows, which were integrated into clinic workflows. Rooming staff reviewed the registry list containing color-coded markers to identify patients who were overdue for one or more components of CHB management. Rooming staff then sent EHR-based messages to primary care clinicians about overdue items and requested that clinicians review patients’ charts and place necessary orders. Rooming staff also communicated with patients directly about being overdue for CHB care.

To compare pilot registry patients to those in usual care, the study sample included patients with CHB seen within the entire HP care system during September 1, 2016–August 31, 2019, who were aged ≥ 18 years as of September 1, 2019. Diagnosis of CHB was defined as the most recent laboratory result for HBsAg or HBV DNA available in the patient’s HP EHR at any time during July 19, 1989–August 31, 2019 showing the presence of HBsAg or detectable HBV DNA. We divided eligible patients into three care groups based on their status as of September 1, 2019: CIH pilot (CIH), gastroenterology (GI), and HP primary care (PC). Patients included in the CIH group had an established primary care clinician at CIH. We defined the GI group as patients who did not have a primary care clinician at CIH and who had been seen by GI at least twice for hepatitis B with the most recent visit occurring within 18 months before September 1, 2019. We included the remaining patients in the PC group.

### Data source.

We used HP administrative and clinical databases composed of patient records from both inpatient and outpatient clinical encounters, laboratory test results, imaging, and other clinical data. Patient demographic information was extracted from HP’s EHR. Diagnoses for hepatitis C infection, HIV infection, cirrhosis, and HCC were identified using ICD-10 codes from a patient’s problem list (no date restrictions) or assigned during any clinical encounter during September 1, 2016–August 31, 2019 (see footnote to Table 1). We categorized patients as “history of CHB treatment” if there was a recorded prescription for at least 1 dose of hepatitis B antiviral medication during the 3 years before the pilot study start (September 1, 2016–August 31, 2019), see Table 1 footnote for list of included medications. Insurance status was based on the most recent insurance listed for a patient as of September 1, 2019.

**Table 1:** Baseline patient demographic information and medical history for patients seen within HealthPartners (HP) care system during September 1, 2016–August 31, 2019

	CIH, *n* = 213	GI,* *n* = 656	PC, *n* = 4003
Age, median (IQR)	46 (34–60)	46 (38–59)	47 (37–59)
Sex, female, no. (%)	86 (40.4)	324 (49.4)	2,109 (52.7)
Race/ethnicity, no. (%)			
Asian	153 (71.8)	394 (60.1)	1,534 (37.8)
Black	58 (27.2)	196 (29.8)	1,536 (38.4)
White	0	48 (7.3)	718 (17.9)
Unknown	0	7 (1.1)	112 (2.8)
Other	2 (0.9)	5 (0.8)	83 (2.1)
Multiple races	0	4 (0.6)	25 (0.6)
Hispanic	0	1 (0.2)	9 (0.2)
American Indian/Alaska Native	0	1 (0.2)	6 (0.2)
Country of origin,† no. (%)	Myanmar 58 (27.2)	U.S. 98 (14.9)	U.S. 829 (20.7)
	Vietnam 40 (18.8)	Vietnam 81 (12.4)	Somalia 329 (8.2)
	Somalia 23 (10.8)	Laos 56 (8.5)	Vietnam 209 (5.2)
	Ethiopia 21 (9.9)	Somalia 46 (7.0)	Laos 193 (4.8)
	Laos 18 (8.5)	Ethiopia 36 (5.5)	Ethiopia 164 (4.1)
	Cambodia 17 (8.0)	China 29 (4.4)	Liberia 163 (4.1)
	Missing 3 (1.4)	Missing 144 (21.9)	Missing 1375 (34.4)
Primary language,† no. (%)	Karen 64 (30.0)	English 428 (65.1)	English 2,722 (68.0)
	Vietnamese 37 (17.4)	Vietnamese 70 (10.7)	Somali 361 (9.0)
	English 33 (15.5)	Hmong 29 (4.4)	Vietnamese 224 (5.6)
	Somali 19 (8.9)	Somali 28 (4.3)	Hmong 144 (3.6)
	Cambodian 15 (7.0)	Cambodian 19 (2.9)	Karen 97 (2.4)
	Oromo 13 (6.1)	Karen 16 (2.4)	Cambodian 79 (2.0)
	Missing 0	Missing 0	Missing 5 (0.1)
Interpreter requested for healthcare encounters, no. (%)	175 (82.2)	200 (30.5)	1,043 (26.1)
Health insurance, no. (%)			
Medicaid	136 (63.8)	247 (37.7)	1,430 (35.7)
Medicare	32 (15.0)	63 (9.6)	417 (10.4)
Private	37 (17.4)	312 (47.6)	1848 (46.2)
Dual Insurance	1 (0.5)	9 (1.4)	35 (0.8)
None	0	1 (0.2)	3 (0.1)
Missing	7 (3.3)	24 (3.7)	270 (6.7)
History of CHB treatment,‡ no. (%)	59 (27.7)	317 (48.3)	324 (8.1)
Most recent HBV DNA > 2,000, no. (%)	36 (16.9)	159 (24.2)	345 (8.6)
Most recent ALT elevated (> 40), no. (%)	39 (18.3)	128 (19.5)	590 (14.7)
Positive HBsAg on file, no. (%)	200 (93.0)	590 (89.9)	2,786 (69.6)
Comorbid conditions,§ no. (%)			
Hepatitis C	3 (1.4)	16 (2.4)	157 (3.9)
HIV	1 (0.5)	10 (1.5)	117 (2.9)
Cirrhosis	12 (5.6)	86 (13.1)	119 (3.0)
Hepatocellular carcinoma	1 (0.4)	11 (1.7)	17 (0.4)

ALT = alanine transaminase; CHB = chronic hepatitis B; CIH = HealthPartners Center for International Health; GI = HealthPartners Gastroenterology; HBsAg = hepatitis B surface antigen; HBV = hepatitis B virus; IQR = interquartile range; PC = HealthPartners Primary Care. Patients were 18 years or older as of September 1, 2019, and met our case definition for chronic hepatitis B (i.e., presence of hepatitis B surface antigen or detectable HBV DNA in most recent laboratory result available in the HP electronic health record during July 19, 1989–August 31, 2019), *N* = 4,872. Data are *n* (%) unless otherwise noted.

* Seen by GI at least two times ever; the most recent visit must have occurred within the 18 months before September 1, 2019.

† Six most common for each group.

‡ Prescription for at least one of the following during September 1, 2016–August 31, 2019: abacavir sulfate-lamivudine, abacavir-lamivudine-zidovudine, adefovir dipivoxil, efavirenz-emtricitabine-tenofovir, emtricitabine, emtricitabine-tenofovir, entecavir, lamivudine, lamivudine-zidovudine, peginterferon alfa-2A, tenofovir alafenamide, or tenofovir disoproxil.

§ International Classification of Diseases (ICD)-9 and ICD-10 codes used for identification of comorbid conditions: hepatitis C (ICD-9 = V02.62, 070.44, 070.51, 070.54, 070.62, 070.70, 070.71; ICD-10 = B17.10, B18.2, B19.20); HIV (ICD-9 = 042; ICD-10 = B20); cirrhosis (ICD-9 = K74.60, K71.7, K74.69; ICD-10 = 571.5); hepatocellular carcinoma (ICD-9 = 155.0; ICD-10 = C22.0).

### Outcomes.

The primary outcomes were alanine transaminase (ALT) and HBV DNA laboratory testing and HCC screening by ultrasound, contrast-enhanced computed tomography, or magnetic resonance study performed within the 12 months before the baseline date (September 1, 2019) and the pilot registry end date (February 28, 2020). Outcomes were evaluated individually and also combined into a bundled measure indicating up-to-date (UTD) status. This definition was chosen for simplicity of registry creation and was based on AASLD guidelines for the management of CHB.^20^ HCC screening was assessed for all patients regardless of race/ethnicity, age, presence of cirrhosis, or family history. Frequency of alpha-fetoprotein (AFP) laboratory testing as an additional HCC screening tool was also assessed but was not used in the UTD definition. For patients who were UTD at baseline, we recorded whether they maintained their UTD status at the end of the pilot. For patients who were not UTD at baseline, we evaluated whether they achieved UTD status by the pilot registry end date. We received unstructured, anecdotal feedback from staff at CIH about utility and feasibility of a clinic-based hepatitis B registry.

### Data analysis.

We provide baseline descriptive information for patient demographics and comorbid conditions and calculate absolute frequencies of individual laboratory testing and liver imaging for each group at baseline and registry end date. We describe and compare rates of laboratory testing and HCC screening between groups. To determine the effectiveness of the registry as compared with usual care, we used 2 × 2 contingency tables to calculate the odds of maintaining or achieving UTD status at the end of the 6-month pilot period for patients in each group. Odds ratios (ORs), 95% confidence intervals (CIs), and χ^2^ tests were used to compare CIH to both the GI and PC groups. Because we used a nonstandard definition for CHB (i.e., most recent laboratory result for HBsAg or HBV DNA showing the presence of HBsAg or detectable HBV), we conducted a sensitivity analysis by limiting included patients to those with confirmed CHB, defined as having at least two laboratory tests positive for either HBsAg or HBV DNA performed at least 6 months apart. Exploratory analysis focused on the CIH group stratified by whether patients received regular care from a liver specialist as defined for the GI group (i.e., seen by GI at least twice for hepatitis B with the most recent visit occurring within the 18 months before September 1, 2019). We applied intent-to-treat methodology in all analyses based on a patient’s baseline care group assignment. All analyses were performed in SAS version 9.4 (SAS Institute Inc., Cary, NC) using two-sided alphas of 0.05.

## RESULTS

We included 4,872 patients divided into groups as follows: 213 in CIH, 656 in GI, and 4,003 in PC. Table 1 provides baseline demographic and medical information for all groups. CIH patients were more likely to be foreign born. The most common country of origin for both GI and PC was the United States. Among the CIH group, 82% of patients required a language interpreter for medical encounters. CIH patients had higher rates of government sponsored health insurance while most patients in GI and PC were privately insured. The prevalence of hepatitis C, HIV, and HCC were similarly low in all groups. Thirteen percent of the GI patients had a previous diagnosis of cirrhosis, which was notably higher than in the two other groups (CIH: 6%, PC: 3%).

### Baseline status.

At baseline, the majority of GI patients were UTD (69%) followed by CIH (51%). Only 11% of patients in PC were UTD at baseline (Table 2). The most common laboratory test performed for all groups was ALT and AFP was the least common. Patients in CIH were more likely to have both AFP results and HCC imaging performed in the past 6 months compared with GI (27 versus 22%, Table 3).

**Table 2:** Percent of patients noted to be up-to-date* for chronic hepatitis B management in three care groups, 1) CIH pilot both as one group and divided into two groups for exploratory analysis based on whether the patients had been seen by a liver specialist (CIH + GI), 2) GI, and 3) HP PC at baseline (September 1, 2019) and pilot registry end date (February 28, 2020); *N* = 4,872

	CIH			
	CIH, *n* = 213	CIH + GI/ CIH no GI, *n* = 73/140	GI, *n* = 656	PC, *n* = 4003
Baseline	51%	81%/35%	69%	11%
Pilot registry end date	62%	79%/54%	59%	10%

CIH = HealthPartners Center for International Health; GI = HealthPartners Gastroenterology; PC = HealthPartners Primary Care.

* Alanine transaminase and hepatitis B virus DNA laboratory testing as well as hepatocellular carcinoma screening by ultrasound, contrast-enhanced computed tomography, or magnetic resonance study performed within the 12 months of selected time points.

**Table 3:** Frequency of individual laboratory tests and liver imaging for three care groups, 1) CIH pilot, 2) GI, and 3) HP PC performed within 6 months prior to hepatitis B registry start (September 1, 2019) or end (February 28, 2020), *N* = 4,872

	6 Months preregistry start (3/1/2019– 8/31/2019), no. (%)	6 Months before registry end (9/1/2019– 2/28/2020), no. (%)
CIH, *N* = 213		
ALT	104 (48.8)	119 (55.9)
HBV DNA	93 (43.7)	109 (51.2)
HCC screening = AFP + imaging	57 (26.8)	72 (33.8)
HCC screening = only AFP	16 (7.5)	13 (6.1)
HCC screening = only imaging	31 (14.6)	36 (16.9)
GI, *N* = 656		
ALT	420 (64.0)	340 (51.8)
HBV DNA	372 (56.7)	285 (43.4)
HCC screening = AFP + imaging	144 (22.0)	101 (15.4)
HCC screening = only AFP	44 (6.7)	29 (4.4)
HCC screening = only imaging	204 (31.1)	163 (24.8)
PC, *N* = 4,003		
ALT	1,098 (27.4)	1,006 (25.1)
HBV DNA	493 (12.1)	399 (10.0)
HCC screening = AFP + imaging	125 (3.1)	92 (2.3)
HCC screening = only AFP	32 (0.8)	55 (1.4)
HCC screening = only imaging	311 (7.8)	262 (6.5)

AFP = alpha-fetoprotein; ALT = alanine transaminase; CHB = chronic hepatitis B; CIH = HealthPartners Center for International Health; GI = HealthPartners Gastroenterology; HBsAg = hepatitis B surface antigen; HBV = hepatitis B virus; HCC = hepatocellular carcinoma surveillance; PC = HealthPartners Primary Care. Data are *n* (%) unless otherwise noted.

### Change at 6 months.

At the end of the pilot, the CIH group had the highest rate of UTD patients, increasing by 11% from baseline. The percent of UTD patients in the GI group decreased by 10% over this same period while the PC group was unchanged. The odds of maintaining UTD status were higher than the odds of achieving UTD status for all three groups (Table 4). For CIH patients who were UTD at baseline, the odds of maintaining UTD status at 6-months were 8.8 compared with 3.3 for GI patients and 1.9 for PC patients. A similar pattern occurred for patients who were not UTD at baseline with the odds of achieving UTD status being highest for CIH followed by GI and PC (0.5, 0.2, 0.04).

**Table 4:** Odds of maintaining UTD* status for patients with chronic HBV infection at 6 months for those who were UTD at baseline and odds of achieving UTD status at 6 months for those who were not UTD at baseline for three groups, 1) CIH pilot, 2) Gastroenterology, and 3) HP PC; *N* = 4,872

Odds of	CIH, *n* = 213	CIH + GI/ CIH no GI, *n* = 73/140	GI, *n* = 656	PC, *n* = 4,003	Odds ratio (95% confidence interval)		
					CIH Pilot vs. GI	CIH Pilot vs. PC	Within CIH: CIH + GI vs. CIH no GI
Maintaining	8.8	6.6 / 15.3	3.3	1.9	2.6† (1.4–5.1)	4.7† (2.5–9.1)	0.4 (0.1–1.7)
Achieving	0.5	1.0 / 0.5	0.21	0.04	2.4† (1.4–4.2)	13.7† (8.8–21.3)	2.1 (0.7–6.7)

CIH = HealthPartners Center for International Health; GI = HealthPartners Gastroenterology; HBV = hepatitis B virus; PC = HealthPartners Primary Care.

* Up-to-date (UTD) = alanine transaminase and HBV DNA laboratory testing as well as hepatocellular carcinoma screening by ultrasound, contrast-enhanced computed tomography, or magnetic resonance study performed within 12 months of selected time points.

† *P* < 0.05 as determined by chi-square test.

Using ORs to formally compare groups (Table 4), the CIH group had significantly higher odds (*P* < .05) of maintaining UTD status compared with GI (OR = 2.6, 95% CI = 1.4, 5.1, χ^2^ = 8.8, *df* = 1, *P* = 0.003) and PC (OR = 4.7, 95% CI = 2.5, 9.1, χ^2^ = 25.1, *df* = 1, *P* < .001). The odds of achieving UTD status for patients who were not UTD at baseline were also significantly higher for CIH patients compared with GI (OR = 2.4, 95% CI = 1.4, 4.2, χ^2^ = 10.9, *df* = 1, *P* < .001) and PC (OR = 13.87, 95% CI = 8.8, 21.3, χ^2^ = 224, *df* = 1, *P* < .001).

### Sensitivity analysis.

Within our sample of 4,872 patients, 51% met the confirmed CHB definition (at least two positive tests for HBsAg and/or HBV DNA at least 6 months apart), with 81% of patients in the CIH group having both required laboratories on file, 94% in HP GI, and 42% in PC. We found the same relationships observed in our primary work among this smaller sample, with CIH seeing a higher rate of UTD status at 6 months than the other two groups (Table 5).

**Table 5:** Sensitivity analysis: percent of confirmed (CHB) patients noted to be UTD* for CHB management in three care groups: 1) CIH pilot, both as one group and divided into two groups for exploratory analysis based on whether the patients had been seen by a liver specialist (CIH + GI); 2) GI; and 3) HP PC at baseline (September 1, 2019) and pilot registry end date (February 28, 2020); *n* = 2,463

	CIH			
	CIH, *n* = 174	CIH + GI/ CIH no GI, *n* = 72/102	GI, *n* = 616	PC, *n* = 1,673
Baseline	57%	81%/40%	71%	16%
Pilot registry end date	67%	79%/59%	60%	15%

CHB = chronic hepatitis B; CIH = HealthPartners Center for International Health; GI = HealthPartners Gastroenterology; HBV = hepatitis B virus; PC = HealthPartners Primary Care.

* Up-to-date (UTD) = alanine transaminase and hepatitis B virus DNA laboratory testing as well as hepatocellular carcinoma screening by ultrasound, contrast-enhanced computed tomography, or magnetic resonance study performed within the 12 months of selected time points.

### Within CIH–GI care versus none.

At baseline, 34% (*n* = 73/213) of the CIH patients also met criteria for being seen by GI (CIH+GI). Of these patients, 81% were UTD at baseline compared with 35% of the CIH patients who did not receive regular GI care. At the end of the pilot registry, the proportion of UTD patients in the CIH+GI group remained constant. For those in the CIH group without regular GI care, the UTD status increased by 19% to 54% (Table 2). The odds of maintaining UTD status were higher in the CIH group without regular GI care, whereas the odds of achieving UTD status was higher in the CIH+GI group. Neither of these differences were statistically significant (Table 4).

### CIH staff feedback.

CIH staff reported difficulty managing the increased workload of the registry in the context of a busy primary care practice with its many competing demands. Although CIH staff were pleased the registry improved care for CHB patients, there was agreement that the increased work required to maintain it, at least in the current form, was not sustainable long-term at the clinic level.

## DISCUSSION

Standardizing care using the hepatitis B pilot registry successfully increased the percentage of patients with CHB who had UTD laboratory surveillance and HCC screening by the definitions we used. HP Center for International Health has 40 years of experience in caring for refugees and immigrants, with a care delivery model based on bilingual, bicultural staff and clinicians, dedicated social work staff, use of professionally trained interpreters and clinicians with expertise in refugee health care. Because of the patient population served at this clinic, staff have substantial experience and familiarity with CHB and are motivated to improve outcomes for patients diagnosed with CHB. All these characteristics likely contributed to both a higher baseline UTD status for patients with CHB and success of the pilot registry.^21^ However, as noted by staff, feasibility of a clinic-level CHB registry is poor.

The number of CHB patients included in the CIH pilot is only a fraction (4%) of the HP system-wide patient population with CHB. The majority of HP patients with CHB managed by primary care outside of CIH received UTD care only 11% of the time. Together, these findings suggest that implementation of a system-wide registry may substantially increase the percent of patients completing recommended CHB follow-up.

Within the CIH group, patients that also received regular GI care (CIH+GI) had a higher rate of being UTD at baseline as well as achieving UTD status at the end of the pilot period. These findings suggest a synergistic effect of receiving care in both primary care and GI. However, patients in the CIH only group had higher odds of maintaining UTD status 6 months after registry implementation, which is consistent with the registry’s effectiveness alone. Wong et al. found that patients with more frequent clinic visits were more likely to undergo regular HCC screening than patients with fewer clinic visits.^9^ It is possible that patients who were UTD at baseline had more frequent clinic visits than patients who were not and were thus more likely to comply with recommended CHB care recommendations. It is also possible that patients in the CIH+GI group have a higher level of awareness and understanding of their HBV infection and thus may be more responsive to follow-up reminders about care recommendations provided through registry outreach. Further research to evaluate predictors of adherence to CHB care recommendations in this population are needed to further refine the hepatitis B registry.

Although we are unaware of other EHR-integrated CHB registries in the United States focused on improving long-term management of CHB infected patients, community-based hepatitis B education campaigns have shown the importance of long-term linkage to care.^22^ Standardized education was not a specific intervention in our study; however, patients’ HBV infection was discussed during outreach efforts and clinic visits. These discussions likely provided patient education about both hepatitis B in general and the need for routine monitoring. As shown by one study in Japan, educational outreach can be performed through other modalities such as paper brochures.^23^ Brochures were mailed annually to HBV-infected patients over an 8-year period, resulting in increasing the proportion of patients seeking specialist consultation as well as regular outpatient follow-up care. Due to the time-intensive nature of the pilot registry resulting in increased workload, future interventions could evaluate the impact of written communication on improving adherence to laboratory monitoring and HCC screening for CHB infected patients, which may decrease the demands on busy primary care clinics.

AASLD guidelines for HCC screening in patients with CHB are limited to high-risk groups including patients with cirrhosis, a family history of HCC, and those meeting certain racial and age categories.^24^ We chose to include HCC screening for all included CHB patients as some studies have shown that strict adherence to HCC screening guidelines for CHB patients may lead to delays in HCC diagnosis for noncirrhotic patients under 40 years of age.^25^ While serum AFP has also been used for HCC screening previously, current recommendations note that although AFP can be used in combination with liver imaging to improve the sensitivity for early detection of HCC, AFP is not recommended as a stand-alone biomarker for HCC screening.^20^ Because of this, we used only imaging studies to evaluate UTD status for HCC screening.

We intentionally did not limit the included cohort to patients who received confirmatory HBV testing to more broadly define the quality of recommended follow-up care for patients with CHB. Sensitivity analysis showed consistency of our results when comparing the odds of maintaining and achieving UTD status between the three groups. Of note, the PC group had the lowest percentage of patients (11%) meeting the confirmed CHB definition. This highlights the need for more standardized follow-up of patients who have a positive HBV screening test, as well as the need for increased clinician education regarding CHB.

This analysis has some limitations. There is always the potential for misclassification bias when using administrative data. However, we attempted to mitigate this misclassification by using inclusion criteria and outcomes that relied on laboratory results. We did not look at adherence to liver biopsy guidelines or testing for coinfection as additional components of appropriate CHB care. Additionally, we did not modify our criteria for laboratory screening based on a patient’s stage of HBV infection because these items were felt to be too stringent for implementation of a population registry. Thus, some included patients should have had more frequent laboratory monitoring than the definition we used for UTD status. Although we reported the number of patients with a prescription for at least one dose of hepatitis B antiviral medication, we did not have a robust way to look at current treatment and did not evaluate appropriateness or duration of treatment.

## CONCLUSION

This project shows that a hepatitis B registry, linked with staff outreach to patients, can assist health systems in improving long-term management of patients with CHB and in doing so may decrease associated health inequities for this patient population. Given the concerns about the feasibility of maintaining a CHB registry within primary care due to competing clinical demands and unequal distribution of CHB-infected patients on primary care patient panels, centralization of such a registry within a single department (e.g., hepatology) may be advantageous. For example, centralization within a hepatology department could address the problem of some primary care clinicians’ lack of expertise in CHB management while taking advantage of specialist knowledge in caring for complex patients. Regardless of how or where within a health system a hepatitis B registry is operationalized, our pilot project suggests its implementation should be strongly considered in any health system committed to improving quality of care for patients with CHB as well as promoting health equity.
